# Effects of an 8-week mental preparation program combining progressive muscle relaxation and mental imagery on self-determined motivation and dynamic postural control in youth female handball players: an experimental study

**DOI:** 10.3389/fpsyg.2026.1902591

**Published:** 2026-08-10

**Authors:** Imen Saket, Chiraz Goumni, Achraf Hammami, Adel Driss, Raouf Hammami, Imen Ben Amar

**Affiliations:** 1 Higher Institute of Sport and Physical Education of Ksar-Said, University of Manouba, Manouba, Tunisia; 2 Research Laboratory “Sport Performance, Health and Society (LR23JS01)” Higher Institute of Sport and Physical Education of Ksar-Said, University of Manouba, Tunis, Tunisia; 3 Tunisian Research Laboratory ‘Sports Performance Optimization’ (CNMSS-LR09SEP01), National Center of Medicine and Science in Sports (CNMSS), Tunis, Tunisia; 4 Department of Physiology, Morehouse School of Medicine, Atlanta, GA, United States

**Keywords:** dynamic balance, handball, mental imagery, progressive muscle relaxation, Self-Determination Theory, youth athletes

## Abstract

**Introduction:**

Mental preparation strategies are widely used in youth sport to enhance both psychological and motor performance; however, the combined effects of progressive muscle relaxation and mental imagery on self-determined motivation and dynamic postural control remain insufficiently explored. This study investigated the effects of an 8-week mental preparation program combining Jacobson’s progressive muscle relaxation and mental imagery in cadet female handball players.

**Methods:**

A quasi-experimental pretest-post-test design with two parallel groups was employed. Thirty-six cadet female handball players were allocated to an experimental group (*n* = 18) and a control group (*n* = 18). Self-determined motivation was assessed using the Sport Motivation Scale (SMS-28), and dynamic postural control was evaluated using the Lower Quarter Y-Balance Test (YBT-LQ) before and after the intervention. The experimental group completed three weekly mental preparation sessions in addition to their regular handball training, whereas the control group continued their usual training program.

**Results:**

Significant improvements in YBT-LQ performance were observed over time in both groups, with greater gains in the experimental group (Time × Group interaction: *p* < 0.001, $\eta_{\text{p}}^{2}$ = 0.37, 95% CI [0.13, 0.57]). Significant Time × Group interactions were also found for intrinsic motivation toward accomplishment (*p* < 0.001, $\eta_{\text{p}}^{2}$ = 0.41, 95% CI [0.16, 0.60]), intrinsic motivation to experience stimulation (*p* < 0.001, $\eta_{\text{p}}^{2}$ = 0.35, 95% CI [0.11, 0.55]), intrinsic motivation to know (*p* < 0.043, $\eta_{\text{p}}^{2}$ = 0.12, 95% CI [0.00, 0.33]), identified regulation (*p* < 0.001, $\eta_{\text{p}}^{2}$ = 0.31, 95% CI [0.08, 0.51]), and external regulation (*p* < 0.001, $\eta_{\text{p}}^{2}$ = 0.55, 95% CI [0.31, 0.70]). The interaction for introjected regulation was small ($\eta_{\text{p}}^{2}$ = 0.02) and did not remain significant after correction. Amotivation decreased over time in both groups without a significant Time ×Group interaction (*p* = 0.097, $\eta_{\text{p}}^{2}$ = 0.079, 95% CI [0.00, 0.25]).

**Discussion:**

An 8-week mental preparation program combining progressive muscle relaxation and mental imagery improved dynamic postural control and promoted more autonomous motivational regulation in cadet female handball players. These findings support the integration of structured mental preparation strategies into youth handball training programs to enhance both psychological and motor performance.

## Introduction

Mental preparation has become an essential component of contemporary athletic training, particularly in youth team sports in which psychological, cognitive, and motor capacities develop concurrently (Li et al., 2025; Memmert et al., 2026). In handball, successful performance depends not only on physical and technical abilities but also on athletes’ capacity to regulate emotions, sustain attentional focus, and make rapid decisions in dynamic and unpredictable game situations (Memmert and König, 2025; Silva, 2006). Recent evidence from systematic reviews and applied sport psychology research further highlights the growing importance of psychological skills training (PST) for enhancing performance, wellbeing, and athlete development in youth sport populations (Thrower et al., 2024a,b).

These demands are particularly relevant during adolescence, a developmental period characterized by substantial maturation of neuromotor, cognitive, emotional, and motivational systems (Gee et al., 2018). Adaptations occurring during this stage may have long-term effects on athletic performance, sport participation, and talent development (Lloyd et al., 2015).

Consequently, interventions targeting both psychological and motor dimensions of performance may be especially beneficial for young athletes undergoing rapid developmental changes.

Among the psychological strategies used in sport, progressive muscle relaxation (PMR) and mental imagery have received considerable attention due to their effectiveness in optimizing performance-related psychological processes (Simonsmeier et al., 2021). PMR involves the systematic contraction and relaxation of muscle groups to reduce physiological tension, enhance self-regulation, and improve stress management (Nair et al., 2024). Mental imagery refers to the cognitive simulation of movements, skills, and competitive scenarios, activating neural networks associated with motor planning and execution, and facilitating motor learning and performance (Simonsmeier et al., 2021). Previous studies have demonstrated that both techniques can reduce competitive anxiety, improve attentional control, strengthen self-confidence, and enhance motor performance in athletes (Junli et al., 2021; Sánchez-Sánchez et al., 2023).

Importantly, there is a growing theoretical basis for combining PMR and mental imagery within integrated psychological skills training programs. PMR may create an optimal psychophysiological state characterized by reduced muscular tension and enhanced attentional readiness, thereby facilitating the effectiveness of subsequent imagery practice (Brunelli et al., 2015; Toussaint et al., 2021). In turn, mental imagery can reinforce motor representations and cognitive rehearsal processes while athletes remain in a relaxed and receptive state. This complementary interaction may maximize both psychological and neuromuscular adaptations through concurrent regulation of arousal and activation of motor-related neural pathways (Brunelli et al., 2015; Toussaint et al., 2021). Previous psychological skills training frameworks suggest that combining relaxation and imagery techniques may produce greater benefits than either strategy alone, although empirical evidence remains limited in youth team-sport populations (Simonsmeier et al., 2021; Warfield et al., 2023).

Beyond performance, mental preparation strategies may also influence athletes’ motivational processes. Self-Determination Theory (SDT) provides a comprehensive framework for understanding motivation in sport, emphasizing the importance of satisfying the psychological needs for autonomy, competence, and relatedness in promoting self-determined motivation (Barbosa Cano and Gomez-Baya, 2025). Athletes with higher self-determined motivation tend to demonstrate greater persistence, enjoyment, engagement, and performance (Rodrigues et al., 2024). Consequently, interventions that strengthen psychological resources and self-regulation capacities may contribute positively to motivational quality in young athletes.

In addition to motivation, neuromuscular control is a critical determinant of performance and injury prevention in handball (Schmidt et al., 2023). The present study focused specifically on adolescent female handball players because this population represents a critical yet relatively underrepresented group in the psychological skills training literature. Female adolescent athletes experience unique developmental, psychosocial, and neuromuscular changes during maturation that may influence motivation, emotional regulation, balance performance, and injury susceptibility (Cumming and Hill, 2026). Furthermore, female handball players exhibit relatively high rates of lower-limb injuries, which have been associated with deficits in neuromuscular function, including impaired balance, proprioception, and movement control (Carrasco-Fernández et al., 2025).

Dynamic postural control is a fundamental component of neuromuscular performance, reflecting the ability to maintain stability while performing functional and sport-specific tasks such as landing, cutting, and rapid changes of direction (Gribble et al., 2012). Deficits in dynamic postural control have been linked to an increased risk of lower-extremity injuries, particularly non-contact knee and ankle injuries, and are considered indicative of impaired sensorimotor and neuromuscular function (McGuine et al., 2000; Hrysomallis, 2007). Moreover, dynamic balance assessments are sensitive to training-induced neuromuscular adaptations and are widely used to evaluate the effectiveness of injury-prevention and performance-enhancement interventions in athletic populations (Plisky et al., 2006; Gribble et al., 2012). Therefore, dynamic postural control was selected as the primary outcome because it represents a clinically relevant indicator of neuromuscular function that is closely associated with injury risk and may be responsive to the present intervention.

Although the individual benefits of relaxation and mental imagery have been extensively documented, evidence regarding their combined effects on motivational and neuromuscular outcomes remains limited, particularly in adolescent female handball players. Addressing this gap may provide valuable insights into the role of integrated mental preparation programs in optimizing both psychological and physical aspects of performance during a critical stage of athlete development.

Therefore, the aim of the present study was to investigate the effects of an 8-week mental preparation program combining Jacobson’s progressive muscle relaxation and mental imagery on self-determined motivation and dynamic postural control in cadet female handball players. Based on previous theoretical and empirical evidence, it was hypothesized that the combined intervention would lead to greater improvements in self-determined motivation and dynamic postural control compared with regular handball training alone.

## Materials and methods

### Study design

This study adopted a quasi-experimental, non-randomized cluster-controlled pretest–posttest design with two parallel groups. Participants were recruited from two female handball clubs competing at a similar competitive level. To avoid contamination between participants and preserve the integrity of the intervention, allocation was performed at the club level rather than through individual randomization, resulting in one club assigned to the experimental group (EG; *n* = 18) and the other to the control group (CG; *n* = 18). Because participants were nested within clubs, this design may introduce intra-cluster similarity related to shared coaching practices, training environment, and team culture.

To reduce potential confounding, both clubs were selected based on comparable competitive level, training structure, and weekly training frequency, and all participants continued their usual handball training program throughout the study period. Although training content and coaching staff could not be strictly standardized due to the ecological (real-world club) setting, both teams followed their established seasonal training plans under the supervision of their respective certified coaches, and no changes were made to training load or coaching routines for the purpose of the study. Baseline comparability between groups was verified for all primary and secondary outcome variables prior to the intervention, with no significant differences observed (*p* > 0.05), indicating initial equivalence between clusters on measured outcomes.

Only the experimental group additionally participated in the 8-week mental preparation program combining Jacobson’s progressive muscle relaxation and mental imagery. Outcome measures were assessed under identical standardized conditions at pre- and post-intervention time points in both groups.

### Participants

Although the sample size was relatively modest, it is comparable to previous experimental studies in youth sport psychology and psychological skills training research. An *a priori* power analysis was conducted using G*Power (version 3.1) (Faul et al., 2009) for a repeated-measures ANOVA (group × time interaction), specifying two groups and the number of measurement occasions used in the present study, with an assumed moderate effect size (f = 0.25), α = 0.05, power (1–β) = 0.80, and an assumed correlation among repeated measures consistent with values commonly reported in similar sport science research. The analysis indicated a minimum required total sample size that was in line with the final sample included in the study.

Accordingly, 36 female cadet handball players voluntarily participated in this study. Participants were recruited from two competitive handball clubs competing at the same league level. All eligible players who met the inclusion criteria were invited to participate, and all invited athletes agreed to take part in the study (no refusals were recorded). The mean age of the participants was 15.8 ± 0.7 years, and the mean weekly training volume was 6.3 ± 1.1 h/week. All participants were registered with a club and had at least 3 years of regular competitive handball experience.

Inclusion criteria were: (i) active participation in official club training and competitions, (ii) at least 3 years of competitive handball experience, and (iii) regular training attendance (≥80% of sessions during the previous season). Exclusion criteria were: (i) a history of lower-limb injury within the previous 6 months that resulted in time loss from training or competition (defined as any musculoskeletal injury leading to at least one missed training session or match), (ii) any diagnosed neurological or musculoskeletal disorder affecting motor performance or balance, and (iii) participation in any structured psychological skills training program within the previous 6 months.

Injury history and eligibility criteria were verified through club medical records and confirmed by team medical staff and coaches. Before study participation, athletes and their parents or legal guardians received written information outlining the study purpose, procedures, potential benefits, and risks. Written informed consent was obtained from parents or legal guardians, and written assent was obtained from all participating athletes after a full explanation of the study.

This study was conducted in accordance with the latest version of the Declaration of Helsinki, and the protocol was approved by the Ethics Committee of the National Centre of Medicine and Science of Sports, Tunis (CNMSS-LR09SEP01) prior to commencement.

### Procedures

All participants completed assessments one week before (pre-test) and 1 week after (post-test) the 8-week intervention period. Testing sessions were conducted at the same time of day (late afternoon, between 16:00 and 18:00) to minimize circadian variation and were performed on a standardized indoor handball court surface. Participants wore their usual sport-specific training footwear during all assessments.

A standardized warm-up protocol was completed prior to testing, consisting of 5 min of light running, followed by dynamic stretching and lower-limb activation exercises. The same testing order was maintained for all participants and both groups, with the Sport Motivation Scale (SMS-28) administered first, followed by the Lower Quarter Y-Balance Test (YBT-LQ).

All assessments were conducted by the same trained evaluators to ensure consistency. However, due to the nature of the intervention, assessors were not blinded to group allocation, which may represent a potential source of bias. Self-determined motivation was assessed using the Sport Motivation Scale (SMS-28). All participants were fluent in the language of the questionnaire, and standardized written and verbal instructions were provided prior to completion to ensure uniform administration conditions.

Internal consistency (Cronbach’s alpha) was calculated for each SMS-28 subscale within the present sample and confirmed acceptable reliability. Dynamic postural control was evaluated using the Lower Quarter Y-Balance Test (YBT-LQ), following standardized testing procedures.

#### Sport motivation scale (SMS-28)

Motivation was assessed using the French version of the Sport Motivation Scale (Échelle de Motivation dans les Sports; EMS-28) developed by Brière et al. (1995) and subsequently validated by Pelletier et al. (1995). Based on Self-Determination Theory (Deci and Ryan, 1980), the questionnaire comprises 28 items distributed across seven subscales representing intrinsic motivation (to know, to accomplish, and to experience stimulation), extrinsic motivation (identified, introjected, and external regulation), and amotivation. Participants responded using a 7-point Likert scale ranging from 1 (“does not correspond at all”) to 7 (“corresponds exactly”). Subscale scores were calculated according to the authors’ recommendations. The SMS-28 has demonstrated satisfactory validity and reliability in athletic populations (Brière et al., 1995; Pelletier et al., 1995).

#### Lower Quarter Y-Balance Test

Dynamic postural control was assessed using the Lower Quarter Y-Balance Test (YBT-LQ), a reliable and valid measure of dynamic balance and lower-limb neuromuscular control (Shaffer et al., 2013; Plisky et al., 2021), performed in accordance with established consensus guidelines for Y-Balance testing procedures. Lower-limb length was measured from the anterior superior iliac spine to the distal aspect of the medial malleolus with participants in a supine position. Limb dominance was defined as the preferred kicking leg, as self-reported by the participant. After a standardized familiarization period of four to six practice trials, participants performed three recorded trials in each reach direction: anterior (ANT), posteromedial (PM), and posterolateral (PL). A trial was considered invalid if the participant: (i) lost balance, (ii) lifted or moved the stance foot from the platform, (iii) failed to maintain hands on the hips, or (iv) did not maintain the reach foot contact while returning to the starting position. Invalid trials were discarded and repeated until three successful trials were obtained for each direction. The maximum reach distance for each direction was used for analysis. All reach distances were normalized to limb length, and the composite score was calculated as follows:


Compositescore(%)=

[(ANT+PM+PL)/(3×limb⁢length)]×100

#### Mental preparation program

In addition to their regular handball training, the experimental group participated in an 8-week mental preparation program combining Jacobson’s progressive muscle relaxation (PMR) and mental imagery. The intervention was conducted three times per week, with each session lasting approximately 25–30 min. The program was designed according to established psychological skills training principles and progressively integrated relaxation and imagery techniques into sport-specific contexts (Weinberg and Gould, 2024). The 8-week duration was selected based on previous psychological skills training interventions demonstrating that periods ranging from 6 to 12 weeks are generally sufficient to facilitate the acquisition, consolidation, and application of mental skills while allowing measurable psychological and performance-related adaptations to occur (Simonsmeier et al., 2021; Weinberg and Gould, 2024). This timeframe is also consistent with psychological skill acquisition frameworks emphasizing progression from guided practice to autonomous use in sport-specific contexts. The intervention consisted of three phases: skill acquisition (weeks 1–3), skill consolidation and contextual integration (weeks 4–6), and application in competition-related situations (weeks 7–8). Each session followed a standardized and scripted structure to ensure consistency across participants. The program was delivered by a certified sport psychologist with experience in psychological skills training interventions in youth sport settings, assisted by a trained researcher in sport science. All sessions were conducted in a quiet, dedicated indoor room at the training facility, immediately before regular on-court handball training sessions to facilitate ecological integration while minimizing fatigue-related interference. This timing was chosen to ensure athletes were in a relatively rested state, which is considered optimal for relaxation and imagery effectiveness. Adherence was monitored throughout the intervention. Attendance was recorded for each session, and overall compliance exceeded 90% in the experimental group. Participants who missed a session were allowed to complete a make-up session within the same week when possible to maintain protocol consistency. For the PMR component (10–12 min), participants were instructed to adopt a comfortable seated or supine position, close their eyes, and focus on diaphragmatic breathing (“inhale slowly through the nose… exhale gently through the mouth”). The relaxation sequence followed a standardized proximal-to-distal progression of major muscle groups: hands and forearms, biceps and triceps, shoulders and neck, facial muscles, chest and back, abdomen, thighs, and calves and feet. Each muscle group was trained using a consistent tension–relaxation cycle: “Tense the muscle group firmly for 5–7 s… hold briefly… now release completely and notice the sensation of relaxation for 10–15 s.” Participants were encouraged to maintain relaxed breathing and focus on the contrast between tension and relaxation throughout the sequence. The mental imagery component (10–15 min) followed a structured progression based on PETTLEP principles (Smith et al., 2007; Holmes and Collins, 2001). Participants began in a relaxed position with attentional focus on breathing (“Bring your attention to your breathing and allow your body to remain relaxed”). Imagery then progressed through: (1) basic skill execution (e.g., “Imagine performing a successful pass with correct technique”), (2) action imagery in game context (e.g., fast break execution), (3) tactical decision-making scenarios, (4) high-pressure competitive situations (e.g., final minutes of a close match), and (5) outcome-focused and emotional regulation imagery (confidence, success, and controlled arousal). Participants alternated between internal (first-person) and external (third-person) perspectives across sessions, with emphasis on multisensory integration (visual, kinesthetic, auditory cues). Each session concluded with a 3–5 min recovery phase, during which participants returned to normal breathing, gradually reoriented attention to the external environment, and opened their eyes when ready. The detailed content and weekly progression of the mental preparation program are presented in Table 1.

**TABLE 1:** Overview of the 8-week mental preparation program implemented in the experimental group.

Week	Progressive muscle relaxation (PMR)	Mental imagery
1	Introduction to PMR in a quiet indoor room: seated/supine position, diaphragmatic breathing (“inhale slowly through the nose… exhale gently through the mouth”), and standardized proximal-to-distal tension–relaxation sequence (hands → arms → shoulders → face → trunk → lower limbs).	Introduction to imagery: guided external (third-person) observation of handball actions combined with basic attentional focus on breathing and relaxation.
2	Full PMR sequence emphasizing sport-relevant muscle groups involved in throwing, jumping, and landing; standardized tension (5–7 s) and relaxation (10–15 s) cycles with breathing control.	Internal (first-person) imagery focusing on kinesthetic sensations and correct execution of basic skills (passing, shooting) under relaxed conditions.
3	Simplified PMR protocol combining shortened muscle groups and controlled breathing to enhance efficiency of self-regulation.	Combined visual–kinesthetic imagery of isolated technical skills with emphasis on movement accuracy and body awareness.
4	Brief (“flash”) PMR (∼5–7 s tension / 10–15 s relaxation per group) for rapid arousal regulation.	Real-time game imagery incorporating environmental cues (teammates, opponents, spatial awareness).
5	PMR integrated with cue words (“relax,” “calm,” “loose”) to facilitate rapid relaxation response.	Semi-functional imagery performed in standing/playing posture using ball handling or simulated sport actions.
6	PMR used post-activation for rapid down-regulation and recovery with emphasis on breathing control.	Tactical imagery focusing on decision-making, anticipation, and problem-solving in offensive and defensive contexts.
7	PMR performed under simulated pressure (time constraints, pre-performance routine conditions).	High-pressure competitive imagery (e.g., final minutes of close match) with emotional regulation focus.
8	Integration of PMR into pre-competition routine combining full-body relaxation and breathing regulation.	Outcome and mastery imagery focusing on successful performance, confidence, and optimal execution under competition conditions.

The intervention lasted 8 weeks and was delivered three times per week in a quiet, dedicated indoor room immediately before regular handball training sessions. Each session lasted approximately 25–30 min and consisted of 10–12 min of PMR, 10–15 min of guided mental imagery, and a 3–5 min recovery phase. All sessions were delivered by a certified sport psychologist assisted by a trained sport science researcher. Attendance was monitored, with compliance exceeding 90%. PMR included standardized breathing instructions and a structured tension–relaxation cycle. Mental imagery followed PETTLEP principles and progressively integrated sensory, emotional, and sport-specific contextual elements. PMR, progressive muscle relaxation.

From a theoretical perspective, the combination of PMR and imagery may be interpreted within Self-Determination Theory (SDT). Mental imagery may enhance perceived competence and self-efficacy by allowing athletes to mentally rehearse successful performance and effective decision-making, while PMR may reduce physiological arousal and anxiety, thereby increasing perceived control and readiness. These processes may facilitate more adaptive motivational regulation, particularly through strengthening autonomous forms of motivation (intrinsic and identified regulation). However, changes in controlled forms of motivation (introjected and external regulation) should be interpreted cautiously, as increases in these dimensions do not necessarily reflect more self-determined motivation.

The control group continued their usual technical, tactical, and physical training routines throughout the study period and did not receive any psychological skills training.

### Statistical analysis

Data are expressed as mean ± standard deviation (SD). Normality of data distribution was assessed using the Shapiro–Wilk test, while homogeneity of variances was verified using Levene’s test. In addition, sphericity was not a concern due to the two-level within-subject factor (Time). All assumptions were met for the applied statistical tests. Baseline characteristics between groups were compared using independent samples *t*-tests for continuous variables and chi-square tests for categorical variables. To examine the effects of the 8-week mental training program on dynamic postural control and motivational regulations, a series of two-way mixed analyses of variance (ANOVA) were performed with Time (pre-test, post-test) as the within-subject factor and Group (experimental, control) as the between-subject factor for each outcome variable. The primary outcome was the composite score of the Lower Quarter Y-Balance Test (YBT), while secondary outcomes included the seven motivational regulations from the Sport Motivation Scale (SMS-28). Given the number of motivational subscales (*n* = 7) and their conceptual interdependence, a Bonferroni correction (adjusted alpha = 0.05/7 = 0.007) was applied for secondary outcomes to reduce the risk of Type I error. Both adjusted and unadjusted interpretations are reported where relevant to preserve transparency in exploratory comparisons. No covariates were included in the analyses. This decision was based on the absence of significant baseline differences between groups and the fact that participants were drawn from the same competitive level with comparable structured training environments. Effect sizes for significant effects were calculated using partial eta-squared ($\eta_{\text{p}}^{2}$), with thresholds of 0.01 (small), 0.06 (medium), and 0.14 (large) according to Cohen (1988). In addition, 95% confidence intervals (95% CI) were computed for mean differences and effect sizes to provide estimates of precision. *Post hoc* pairwise comparisons were conducted to decompose significant Time × Group interactions, with Bonferroni adjustment applied swhere appropriate. The significance level was set at *p* < 0.05 for the primary outcome. For secondary outcomes, adjusted significance levels were applied as described above. Statistical analyses were performed using IBM SPSS Statistics (version 26.0; IBM Corp., Armonk, NY, United States), and confidence intervals were calculated using standard SPSS procedures.

## Results

### Assumption testing and baseline comparisons

The descriptive statistics, *p*-values, effect sizes, and confidence intervals for all outcome variables are presented in Table 2. Normality of data distribution was confirmed for all study variables using the Shapiro–Wilk test (*p* > 0.05), and homogeneity of variances was verified using Levene’s test (*p* > 0.05). All assumptions for parametric analyses were therefore satisfied. Baseline comparisons indicated no significant differences between the experimental and control groups for demographic variables or for the Y-Balance Test and motivational regulation subscales (all *p* > 0.05), confirming initial group equivalence.

**TABLE 2:** Effects of the intervention on dynamic postural control and motivation variables.

Variables	Experimental group		Control group		*P*-value			Partial eta squared (η*p*^2^)		
	Pre-test (M ± SD)	Post-test (M ± SD)	Pre-test (M ± SD)	Post-test (M ± SD)	Time	Group	Time × Group	Time	Group	Time × Group
Y-Balance test	105.67 ± 6.34	107.15 ± 6.29	101.28 ± 5.57	104.32 ± 5.72	<0.001	<0.001	<0.001	0.50	0.16	0.37
Intrinsic motivation—to know	15.44 ± 1.65	18.56 ± 2.35	14.61 ± 2.45	16.00 ± 2.56	<0.001	0.12	<0.001	0.46	0.17	0.11
Intrinsic motivation—toward accomplishment	16.61 ± 2.59	20.39 ± 3.23	15.83 ± 2.68	16.33 ± 2.40	<0.001	0.008	<0.001	0.54	0.19	0.40
Intrinsic motivation—to experience stimulation	17.56 ± 2.83	20.94 ± 2.26	17.22 ± 2.75	18.25 ± 3.60	0.156	<0.001	<0.001	0.058	0.37	0.34
Extrinsic motivation—identified regulation	15.67 ± 2.49	19.44 ± 2.45	15.33 ± 2.91	16.39 ± 2.95	<0.001	0.042	<0.001	0.58	0.11	0.30
Extrinsic motivation—introjected regulation	16.94 ± 1.55	18.72 ± 1.48	14.56 ± 2.17	16.94 ± 2.12	<0.001	<0.001	<0.001	0.52	0.32	0.023
Extrinsic motivation—external regulation	16.78 ± 1.89	12.94 ± 1.58	16.56 ± 2.38	15.78 ± 2.32	<0.001	0.044	<0.001	0.73	0.11	0.54
Amotivation	13.33 ± 2.87	9.50 ± 2.03	13.22 ± 2.13	10.72 ± 2.37	<0.001	0.42	0.097	0.65	0.019	0.079

Values are presented as mean ± standard deviation (M ± SD). *P*-values and partial eta squared (η^2^) were obtained from the repeated-measures ANOVA. The effects of Time, Group, and Time × Group interaction are reported. *P*-values are reported for the main effects of Time, Group, and Time × Group interaction.

### Effects of the mental preparation program on dynamic postural control

**FIGURE 1:**
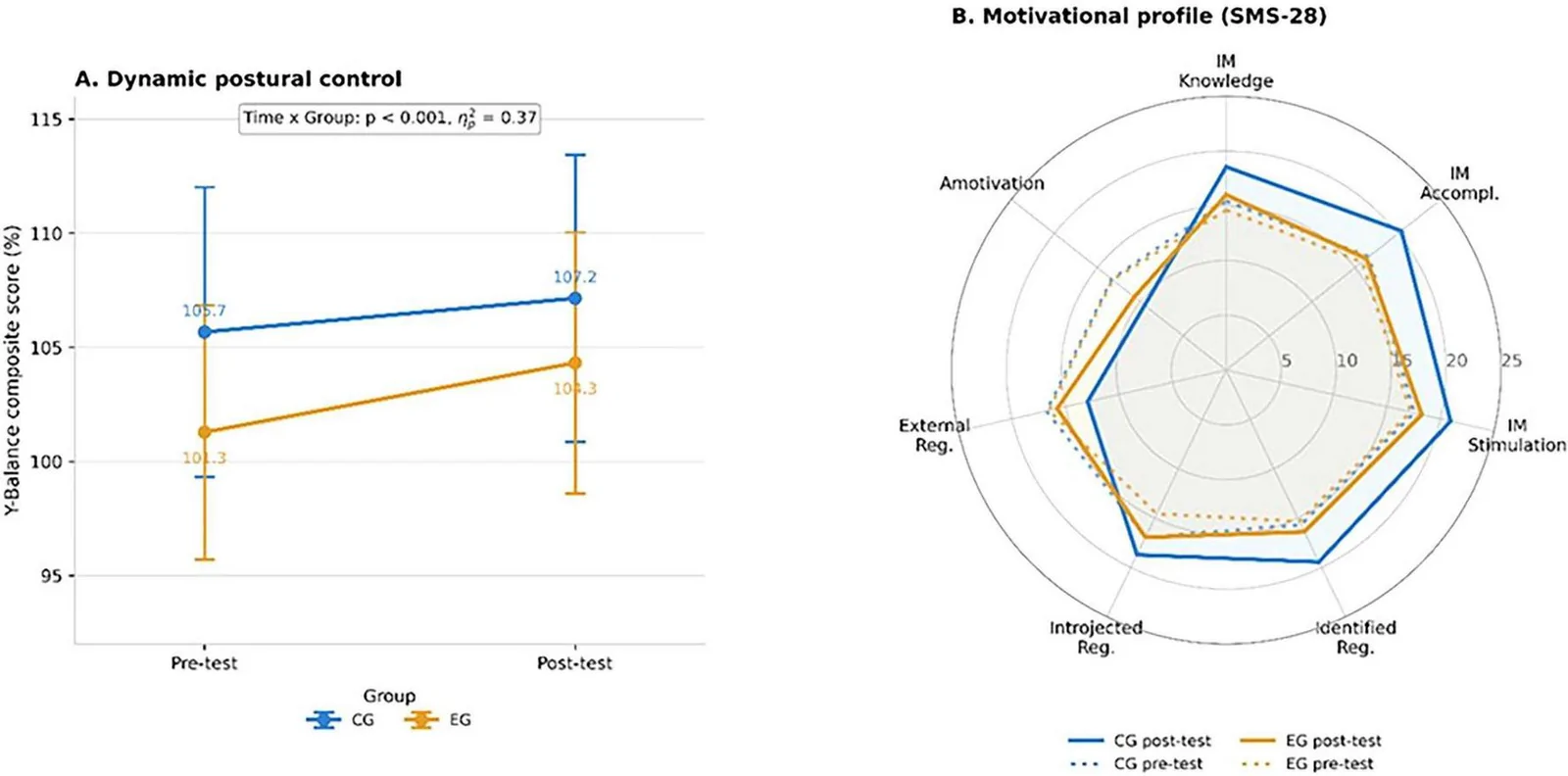
Effects of the 8-week mental preparation program on dynamic postural control and motivational profile in cadet female handball players. **(A)** Changes in dynamic postural control assessed by the Lower Quarter Y-Balance Test (YBT-LQ) composite score (%) from pre-test to post-test in the experimental group (EG) and control group (CG). The Time × Group interaction was significant (*p* < 0.001, $\eta_{\text{p}}^{2}$ = 0.37). **(B)** Changes in the motivational profile assessed by the Sport Motivation Scale (SMS-28), including intrinsic motivation (knowledge, accomplishment, and stimulation), identified regulation, introjected regulation, external regulation, and amotivation, before and after the intervention in both groups. Values are presented as mean ± standard deviation.

Results of the two-way mixed ANOVA for the Y-Balance Test are presented in Figure 1A and Table 3. A significant main effect of time was observed (*p* < 0.001, $\eta_{\text{p}}^{2}$ = 0.51, 95% CI [0.27, 0.67]), indicating overall improvement in dynamic postural control from pre-test to post-test across both groups. A significant main effect of group was also found (*p* = 0.016, $\eta_{\text{p}}^{2}$ = 0.16, 95% CI [0.01, 0.38]). Importantly, the Time × Group interaction was significant (*p* < 0.001, $\eta_{\text{p}}^{2}$ = 0.37, 95% CI [0.13, 0.57]), indicating greater improvements in the experimental group compared with the control group.

**TABLE 3:** Characteristics of the study participants (cadet female handball players, *N* = 36).

Variable	Mean ± SD/description
Age (years)	15.8 ± 0.7
Training volume (h/week)	6.3 ± 1.1
Sex	Female
Sport	Handball
Competitive level	Cadet (youth)
Club affiliation	Registered competitive club players
Handball experience (years)	≥3 years
Inclusion criteria	Regular training attendance; participation in official competitions; ≥3 years of experience; no injury or neurological disorder affecting performance
Ethics approval	Approved by CNMSS-LR09SEP01 (National Centre of Medicine and Science of Sports, Tunis)
Consent	Written informed consent obtained from parents/legal guardians and participants
Ethical compliance	Declaration of Helsinki

Values are presented as mean ± standard deviation (M ± SD) for continuous variables and as categorical descriptions for qualitative variables. The table summarizes demographic and sport-specific characteristics of cadet female handball players, including age, training load, competitive experience, and eligibility criteria. It also reports ethical considerations related to study approval, informed consent, and compliance with the Declaration of Helsinki. CNMSS-LR09SEP01 refers to the Ethics Committee of the National Centre of Medicine and Science of Sports, Tunis.

Descriptive statistics showed that the experimental group improved from 105.67 ± 6.35 to 107.15 ± 6.04, whereas the control group showed a smaller change from 101.29 ± 5.57 to 101.49 ± 5.73.

### Effects of the mental preparation program on self-determined motivation

The changes in motivational regulation across the intervention are illustrated in Figure 1B, whereas the detailed statistical results are presented in Table 2. Given the analysis of seven interrelated motivational subscales, a Bonferroni correction was applied (adjusted α = 0.007). Results are reported accordingly, with both unadjusted and corrected interpretations where relevant. Confidence intervals for effect sizes are presented in Table 3.

### Intrinsic motivation

For intrinsic motivation to know, a significant main effect of time was observed (*p* < 0.001, $\eta_{\text{p}}^{2}$ = 0.47, 95% CI [0.22, 0.64]). The group effect was significant (*p* = 0.012, $\eta_{\text{p}}^{2}$ = 0.17, 95% CI [0.01, 0.39]), while the Time × Group interaction was also significant (*p* = 0.043, $\eta_{\text{p}}^{2}$ = 0.12, 95% CI [0.00, 0.33]), although this interaction did not remain robust after Bonferroni correction.

Intrinsic motivation toward accomplishment showed significant effects of time (*p* < 0.001, $\eta_{\text{p}}^{2}$ = 0.54, 95% CI [0.30, 0.69]), group (*p* = 0.008, $\eta_{\text{p}}^{2}$ = 0.19, 95% CI [0.02, 0.41]), and a significant Time × Group interaction (*p* < 0.001, $\eta_{\text{p}}^{2}$ = 0.41, 95% CI [0.16, 0.60]), which remained significant after correction. The experimental group increased from 16.61 ± 2.59 to 20.39 ± 3.24, whereas the control group showed a slight increase from 15.83 ± 2.68 to 16.33 ± 2.40.

For intrinsic motivation to experience stimulation, no significant time effect was observed (*p* = 0.157, $\eta_{\text{p}}^{2}$ = 0.06, 95% CI [0.00, 0.26]). However, significant group (*p* < 0.001, $\eta_{\text{p}}^{2}$ = 0.38, 95% CI [0.13, 0.57]) and Time × Group interaction effects (*p* < 0.001, $\eta_{\text{p}}^{2}$ = 0.35, 95% CI [0.11, 0.55]) were found, indicating intervention-related changes despite the non-significant overall time effect.

### Extrinsic motivation

Identified regulation showed significant effects of time (*p* < 0.001, $\eta_{\text{p}}^{2}$ = 0.58, 95% CI [0.35, 0.72]), group (*p* = 0.042, $\eta_{\text{p}}^{2}$ = 0.12, 95% CI [0.00, 0.33]), and a significant Time × Group interaction (*p* < 0.001, $\eta_{\text{p}}^{2}$ = 0.31, 95% CI [0.08, 0.51]), with the interaction remaining significant after Bonferroni adjustment.

Introjected regulation demonstrated significant effects of time (*p* < 0.001, $\eta_{\text{p}}^{2}$ = 0.53, 95% CI [0.28, 0.68]), group (*p* < 0.001, $\eta_{\text{p}}^{2}$ = 0.32, 95% CI [0.09, 0.53]), and a marginal but very small interaction effect (*p* = 0.02, $\eta_{\text{p}}^{2}$ = 0.02, 95% CI [0.00, 0.07]). This interaction did not survive Bonferroni correction, suggesting limited intervention specificity for this subscale.

External regulation decreased significantly over time (*p* < 0.001, $\eta_{\text{p}}^{2}$ = 0.73, 95% CI [0.56, 0.83]), with a non-significant group effect (*p* = 0.044, $\eta_{\text{p}}^{2}$ = 0.11, 95% CI [0.00, 0.33]) and a significant Time × Group interaction (*p* < 0.001, $\eta_{\text{p}}^{2}$ = 0.55, 95% CI [0.31, 0.70]), indicating a stronger reduction in the experimental group (16.78 ± 1.90–12.94 ± 1.59).

### Amotivation

Amotivation decreased significantly over time (*p* < 0.001, $\eta_{\text{p}}^{2}$ = 0.65, 95% CI [0.40, 0.78]), indicating a general reduction across both groups. However, neither the group effect (*p* = 0.42, $\eta_{\text{p}}^{2}$ = 0.019, 95% CI [0.00, 0.20]) nor the Time × Group interaction (*p* = 0.097, $\eta_{\text{p}}^{2}$ = 0.079, 95% CI [0.00, 0.25]) reached statistical significance, suggesting that the reduction was not specifically attributable to the intervention.

## Discussion

The present study examined the effects of an 8-week mental preparation program combining Jacobson’s progressive muscle relaxation (PMR) and mental imagery on dynamic postural control and self-determined motivation in cadet female handball players. Overall, athletes who participated in the psychological skills program demonstrated greater improvements in dynamic balance and motivational regulation than those who completed regular handball training alone. These findings suggest that the addition of structured psychological preparation may complement conventional training by influencing both neuromuscular and psychological outcomes. Nevertheless, because the intervention combined multiple psychological techniques and did not include an active control condition, the observed effects should not be attributed exclusively to PMR or mental imagery, as non-specific factors such as increased attention, participant expectations, and additional contact time may also have contributed.

### Dynamic postural control

The results revealed significant time effects and a group × time interaction for Y-Balance Test performance, indicating greater improvements in the experimental group. However, the absolute magnitude of change was relatively modest, and improvements were also observed in the control group. Consequently, although the statistical findings support an intervention effect, the practical significance of these changes should be interpreted cautiously, particularly because minimal clinically important differences for this population have not yet been established.

The observed improvements are nevertheless consistent with previous research indicating that mental imagery can positively influence motor coordination, postural regulation, and movement execution (Nicholson et al., 2019). Contemporary neurocognitive models suggest that imagery activates neural networks overlapping with those involved in actual movement execution, including premotor, supplementary motor, parietal, and cerebellar regions, thereby strengthening internal movement representations and sensorimotor integration (Munzert et al., 2009; Guillot and Collet, 2021). Recent evidence further indicates that imagery-based practice may enhance predictive motor control and the efficiency of perception–action coupling, processes that are fundamental for dynamic balance and postural adjustments during sport-specific movements (Di Rienzo et al., 2023).

Progressive muscle relaxation may have complemented these adaptations by reducing excessive muscular tension and improving autonomic regulation. Lower physiological arousal can facilitate attentional control and movement precision, thereby supporting more effective postural strategies during balance tasks (Laborde et al., 2023). The combination of imagery and relaxation may therefore create favorable psychophysiological conditions for motor learning and postural control by simultaneously enhancing motor representation and optimizing self-regulation processes. Such adaptations may be particularly relevant in handball, where athletes are repeatedly exposed to demanding balance challenges during jumps, landings, and rapid changes of direction (Hammami et al., 2019; Herrington et al., 2021; Timmerman et al., 2024). Nevertheless, these mechanisms remain speculative because no physiological or neurocognitive measures were collected to directly test them.

### Motivational outcomes

The intervention also produced meaningful changes in motivational regulation, indicating that the benefits of the psychological skills program extended beyond neuromuscular adaptations. Significant Time × Group interactions were observed for several dimensions of intrinsic motivation, particularly intrinsic motivation toward accomplishment and intrinsic motivation toward knowledge. These findings suggest that athletes who participated in the intervention became increasingly motivated by opportunities to learn, improve, and master new skills, rather than solely by external rewards or performance outcomes. Such changes are particularly important during adolescence, a developmental period in which motivation can fluctuate considerably due to academic, social, and competitive pressures.

From the perspective of Self-Determination Theory (SDT), intrinsic motivation represents the most self-determined form of behavioral regulation and is generally associated with greater persistence, enjoyment, wellbeing, and long-term sport participation (Ryan and Deci, 2000, 2017). The observed increases in intrinsic motivation may indicate that the intervention contributed to the satisfaction of basic psychological needs, particularly competence and autonomy. Mental imagery may have facilitated this process by enabling athletes to mentally rehearse successful actions and performance scenarios, thereby reinforcing perceptions of effectiveness and mastery. Repeated experiences of imagined success may strengthen athletes’ confidence in their abilities, enhance self-efficacy, and increase willingness to invest effort in training and skill acquisition.

Furthermore, imagery-based practice may encourage athletes to adopt a more process-oriented approach to performance by directing attention toward skill execution, tactical understanding, and personal improvement rather than solely toward competitive outcomes. Such a focus on mastery and learning is consistent with the observed increases in intrinsic motivation toward accomplishment and knowledge and may contribute to more adaptive motivational patterns over time.

The intervention also influenced extrinsic forms of motivation. Identified and introjected regulation increased to a greater extent in the experimental group, whereas external regulation decreased significantly. This pattern is noteworthy because it suggests a progressive movement along the self-determination continuum toward more internalized forms of motivation. Athletes increasingly appeared to engage in training because they personally valued its importance (identified regulation) and because participation became more integrated with their sense of self and personal standards (introjected regulation), rather than because of external pressures, rewards, or obligations.

Several mechanisms may explain these findings. Psychological skills training may enhance self-awareness and self-regulatory capacities by helping athletes better understand and manage their thoughts, emotions, and behavioral responses. In particular, PMR may provide athletes with practical tools for controlling physiological arousal and coping with stress-related symptoms commonly experienced during training and competition. Improved emotional regulation may increase feelings of personal control and autonomy, both of which are central determinants of self-determined motivation. Athletes who perceive themselves as capable of managing performance-related challenges may be more likely to internalize training goals and develop stronger personal commitment to sport participation.

The motivational benefits of the intervention may also reflect broader improvements in psychological functioning. Previous research has shown that psychological skills training can enhance confidence, emotional regulation, attentional focus, and coping effectiveness, all of which are positively associated with self-determined forms of motivation. By fostering a greater sense of competence and perceived control, mental preparation programs may create a motivational environment that encourages sustained engagement and investment in the training process. These interpretations are consistent with contemporary SDT frameworks suggesting that interventions promoting self-regulation and psychological competence can facilitate motivational internalization and support long-term behavioral persistence (Ryan and Deci, 2024).

In contrast, amotivation decreased over time in both groups, with no significant interaction effect. This finding suggests that reductions in amotivation were likely influenced by factors common to both groups rather than by the intervention itself. Regular participation in organized handball training, ongoing involvement in a competitive team environment, and natural seasonal adaptations may have contributed to maintaining athletes’ interest and commitment to sport. It is also possible that baseline levels of amotivation were relatively low, limiting the potential for differential intervention effects. Consequently, while the psychological skills program appeared effective in enhancing more self-determined forms of motivation, its specific influence on reducing amotivation remains less clear and warrants further investigation.

Collectively, these findings suggest that structured psychological skills training may play an important role in supporting motivational development during adolescence. By strengthening perceptions of competence, autonomy, and self-regulation, interventions incorporating relaxation and imagery techniques may help young athletes develop more adaptive motivational profiles, which are known to be associated with greater persistence, enjoyment, performance, and long-term engagement in sport.

### Practical implications

The present findings suggest that structured psychological skills training may represent a valuable complement to traditional physical, technical, and tactical preparation in adolescent handball players. The observed improvements in dynamic postural control and motivational regulation indicate that psychological preparation should not be viewed solely as a tool for enhancing mental wellbeing or managing competitive stress, but rather as an integral component of athlete development that may contribute to both psychological and physical performance outcomes.

From an applied perspective, the intervention was relatively simple, time-efficient, and easy to implement within an existing training framework. Brief sessions combining progressive muscle relaxation and mental imagery can be incorporated before, during, or after regular practice sessions without substantially increasing training load or disrupting technical and tactical activities. Such an approach may be particularly attractive for youth sport settings, where coaches often face constraints related to training time, facility availability, and athlete workload.

The findings are especially relevant during adolescence, a developmental period characterized by rapid biological maturation, ongoing neuromuscular development, and significant psychological changes. During this stage, young athletes frequently experience fluctuations in confidence, motivation, emotional regulation, and attentional control. Psychological skills training may help athletes develop greater self-awareness and self-regulatory capacities, enabling them to better manage the physical and psychological demands associated with training and competition. By promoting more self-determined forms of motivation, these interventions may also contribute to sustained sport participation, greater training engagement, and reduced risk of motivational decline during critical stages of athletic development.

From a performance perspective, improvements in dynamic postural control may have practical relevance for handball, a sport that requires athletes to maintain stability and movement efficiency during jumping, landing, cutting, accelerating, decelerating, and contact situations. Although the magnitude of the observed improvements should be interpreted cautiously, enhanced balance and postural regulation may support more effective execution of sport-specific skills and potentially contribute to safer movement patterns during training and competition.

The motivational findings also have important implications for coaching practice. The observed increases in intrinsic motivation and more self-determined forms of extrinsic motivation suggest that psychological skills training may foster a more positive motivational climate by reinforcing athletes’ perceptions of competence, autonomy, and personal control. Coaches may therefore consider integrating structured mental preparation strategies not only to enhance performance readiness but also to support long-term athlete development and enjoyment of sport.

Taken together, the results support a holistic approach to youth athlete development in which psychological skills training is integrated alongside physical, technical, and tactical preparation. Such an approach may help optimize both performance-related and psychosocial outcomes, thereby contributing to the development of more resilient, motivated, and well-prepared young athletes.

### Limitations

Several limitations should be acknowledged when interpreting the present findings. First, the study employed a quasi-experimental design with club-level allocation rather than individual randomization. Although this approach was selected to minimize contamination between participants, it may have introduced confounding influences related to coaching practices, training culture, team dynamics, and environmental factors that were not fully controlled. Consequently, some of the observed between-group differences may reflect contextual characteristics of the participating clubs rather than the intervention alone.

Second, the absence of an active control condition limits the ability to attribute the observed adaptations specifically to the mental preparation program. Participants in the experimental group received additional structured sessions, which may have generated non-specific benefits associated with increased attention from researchers, greater social interaction, enhanced motivation, or expectancy effects. Therefore, the contribution of progressive muscle relaxation and mental imagery cannot be isolated with certainty.

Third, the relatively small sample size and the inclusion of only two clusters limit the generalizability of the findings and may have reduced the precision of the estimated intervention effects. Because only two teams were included, it was not possible to appropriately model intra-cluster correlation or to statistically separate intervention effects from potential team-level influences. Furthermore, the a priori power analysis was conducted without accounting for clustering effects, which may have resulted in an overestimation of statistical power and an increased risk of imprecise effect estimates.

Additional methodological limitations should also be considered. Assessors were not blinded to group allocation due to practical constraints, creating the possibility of measurement bias. Although standardized testing procedures were implemented to minimize this risk, unconscious expectations may have influenced data collection or participant interactions during testing sessions.

Another limitation concerns the outcome measures selected. Dynamic postural control was assessed using the Y-Balance Test, which, although widely accepted as a valid and reliable measure of dynamic balance, may not fully capture the complex movement demands encountered during handball competition. Similarly, motivation was assessed through self-report questionnaires, which are inherently susceptible to social desirability bias, response bias, and fluctuations in participant interpretation. The inclusion of complementary objective and observational measures may have provided a more comprehensive understanding of the intervention’s effects.

Furthermore, the study did not assess potential mediating variables such as imagery ability, psychological skill acquisition, attentional control, perceived stress, physiological arousal, or autonomic regulation. Consequently, the mechanisms responsible for the observed improvements remain speculative and cannot be directly verified. The absence of these measures also prevents determination of whether changes in motivational regulation and postural control were mediated by improvements in psychological functioning.

The ecological validity of the findings is another important consideration. Although improvements were observed in laboratory- and field-based assessments, no sport-specific performance indicators or competitive outcomes were collected. Therefore, it remains unclear whether the observed benefits translated into enhanced match performance, technical execution, tactical decision-making, injury risk reduction, or other meaningful indicators of sporting success.

Finally, no follow-up assessments were conducted after completion of the intervention. As a result, the durability of the observed adaptations and their long-term impact on athletic development remain unknown. It is therefore unclear whether the benefits persisted beyond the intervention period or whether ongoing psychological skills practice would be required to maintain the observed improvements.

### Future directions

Future research should employ randomized controlled trials involving larger samples and multiple teams to improve statistical power, enhance external validity, and allow appropriate modeling of clustering effects. The inclusion of active control conditions receiving comparable attention and contact time would help isolate the specific contributions of psychological skills training from non-specific intervention effects such as expectancy, social interaction, and increased coach–athlete engagement.

Given the multifaceted nature of the present intervention, future studies should also seek to disentangle the individual and combined effects of progressive muscle relaxation and mental imagery through factorial or multi-arm experimental designs. Such approaches would provide greater insight into whether these techniques exert independent, additive, or synergistic effects on neuromuscular and psychological outcomes.

To better understand the mechanisms underlying observed adaptations, future investigations should incorporate physiological, neurocognitive, and psychophysiological measures. Potential markers may include heart rate variability, electromyographic activity, stress-related biomarkers, attentional control assessments, and measures of imagery ability. The integration of these variables would help clarify the pathways through which psychological skills training influences motor performance, balance regulation, and motivational functioning.

Further research should also extend outcome assessments beyond laboratory-based measures by including sport-specific indicators of performance, such as jump-landings, change-of-direction tasks under decision-making demands, technical efficiency, competitive performance statistics, and injury-related outcomes. Such measures would provide greater ecological validity and help determine whether improvements observed in controlled testing environments translate into meaningful benefits during training and competition.

Additionally, future studies should examine potential moderating factors, including age, biological maturation, competitive level, psychological profile, and previous experience with mental skills training. Identifying which athletes benefit most from these interventions could facilitate the development of more individualized and developmentally appropriate psychological preparation programs.

Finally, longitudinal investigations incorporating follow-up assessments across competitive seasons are needed to determine the durability of intervention-related adaptations and their influence on long-term athletic development. Exploring the optimal frequency, duration, and timing of psychological skills training within annual training plans would further assist coaches and practitioners in maximizing the effectiveness and sustainability of these interventions.

## Conclusion

The present study demonstrates that an 8-week mental preparation program combining Jacobson’s progressive muscle relaxation and mental imagery can produce meaningful improvements in both dynamic postural control and self-determined motivation in cadet female handball players. The observed enhancements in Y-Balance Test performance suggest that psychological skills training may contribute not only to mental readiness but also to functional neuromuscular adaptations relevant to sport-specific performance. In parallel, the positive shifts toward more self-determined forms of motivation highlight the role of structured mental training in supporting athletes’ psychological development during a critical stage of sport specialization.

From a practical perspective, these findings support the integration of brief, structured mental preparation sessions within youth handball training programs. Coaches and sport practitioners can implement progressive muscle relaxation as a rapid tool for arousal regulation and recovery, while mental imagery can be used to reinforce technical learning, improve decision-making, and enhance confidence in competitive situations. The relatively short duration and low-cost nature of the intervention also make it suitable for routine use in club settings without requiring major modifications to existing training schedules.

Future research should extend these findings by examining larger and more diverse samples, including male athletes and different competitive levels, to improve generalizability. Longitudinal studies are also needed to determine the long-term effects of combined mental preparation on performance stability, injury prevention, and athlete development across competitive seasons. Additionally, future work should incorporate objective competitive performance indicators and explore the individual contribution of each component (progressive muscle relaxation versus mental imagery) to better isolate their specific effects and optimize intervention design.

## Data Availability

The raw data supporting the conclusions of this article will be made available by the authors, without undue reservation.
